# Reducing the Cancer Burden of Lifestyle Factors: Opportunities and Challenges of the Internet

**DOI:** 10.2196/jmir.7.3.e26

**Published:** 2005-07-01

**Authors:** Amanda L Graham, David B Abrams

**Affiliations:** ^2^Brown UniversityDepartment of Community HealthProvidence, RIUSA; ^1^Brown Medical SchoolDepartment of Psychiatry and Human BehaviorProvidence, RIUSA

**Keywords:** Cancer, Internet, behavior, lifestyle interventions, transdisciplinary, dissemination

## Abstract

This paper focuses on the Internet as a tool for enhancing behavior and lifestyle changes to reduce the burden of cancer at a population level. The premise of this paper is that the Internet can and should be leveraged to bridge the chasm between basic science, clinical trials, and public health. Our focus is specifically on the opportunity to disseminate effective behavioral science interventions via the Internet in order to decrease the prevalence of behavioral risk factors for cancer. The examples herein are primarily drawn from tobacco use to illustrate issues that can be applied more generally to other behavioral risk factors for cancer. Four areas will be addressed: (1) the scientific basis and rationale for delivering lifestyle behavior change interventions via the Internet; (2) the need to determine the quality of Internet interventions; (3) methodological considerations in conducting evaluations of Internet interventions; and (4) recommendations for a transdisciplinary approach to Internet intervention development and evaluation.

## Cancer and Behavior

Cancer is the second leading cause of death in the United States, accounting for 23% of all deaths [1]. In 2004, approximately 563700 people were expected to die of cancer, and the overall costs for cancer in 2003 have been estimated at $189.5 billion [2]. Behavior plays a key role in many aspects of cancer from prevention through treatment through survivorship [3]. Specifically, tobacco use, poor diet, physical inactivity, alcohol abuse, overexposure to sunlight, and risky sexual activity are associated with 50% to 70% of all cancers [4]. Translated to actual numbers, between 281000 and 395000 cancer deaths each year are entirely preventable. Tobacco use is the largest contributor to the cancer burden, accounting for one third of all cancer deaths and 87% of lung cancer deaths each year [2].

### Prevalence of Behavioral Risk Factors

Millions of Americans continue to engage in risky behaviors and fail to proactively adopt protective behaviors for cancer. Approximately 23% of adults smoke [2]. Less than one in four adults eats the recommended servings of fruits and vegetables, and about 38% of all adults do not engage in any physical activity during their leisure time [2]. The prevalence of obesity has increased to 28% for men and 33% for women [2].

In addition to engaging in behaviors that put them at risk for cancer, many adults do not follow recommended cancer screening guidelines. Cancer screening has been shown to reduce mortality from cancers of the breast, uterine cervix, colon, and rectum through early detection and treatment [2]. Yearly mammograms are recommended for all women beginning at age 40, yet the prevalence of mammography in women 40 years and older was only 62% in 2000 [5]. Annual fecal occult blood tests and flexible sigmoidoscopy every five years are recommended for colorectal cancer screening for adults age 50 and over. In 2002, only 22% of age-appropriate adults received a fecal occult blood test, and 41% underwent flexible sigmoidoscopy [5].

### The Need for Improved Dissemination

Significant reductions in the burden of cancer are possible through changes in health behaviors. It has been estimated that the rate of new cancer cases would decline by 19% and the rate of cancer deaths would decline by 29% if proven behavior change interventions were put into practice [6]. Effective and rigorously tested interventions do exist for reducing tobacco use [7], increasing physical activity [8], reducing sun exposure [9], and reducing alcohol misuse [10]. Although these interventions have been effective in producing meaningful (and at times sustainable) behavior change in clinical trials, they need to be proactively marketed, disseminated, and made accessible on a much larger scale if they are to make a population impact on cancer. The impact of behavioral interventions on cancer prevention and control is limited by the failure to transfer evidence-based findings into the widespread delivery of both individual and population health care. It has been estimated that Americans receive only about half of recommended medical care [11]. As stated in a recent report by the Institute of Medicine, “The American health care delivery system is in need of fundamental change.... The care delivered is not, essentially, the care we should receive.... Between the health care we have and the care we could have lies not just a gap, but a chasm...” [12] (p. 1).

## Opportunities of the Internet for Cancer Prevention and Control

### Consumer Demand for Online Health Information

The Internet may be the most important dissemination vehicle to improve individual and overall public health at reasonable societal cost. Breakthroughs in informatics and computer technology come at an opportune time to advance individual level behavior change on a population-wide basis. With thousands of health-related Web sites in existence, the Internet now plays a meaningful role in the health care system and is increasingly available to those with lower incomes and education [13]. Approximately 80% of adult Internet users (estimated at 93 million Americans) have searched for health information [14]. The majority looks for information on a specific disease or condition, and many users report looking for information related to lifestyle behavior change: 36% have searched for information on exercise or fitness, 10% for sexual health information, and 6% for information on how to quit smoking [14]. Six percent of 93 million translates to more than 5.5 million individuals who have looked for smoking cessation information. Not surprisingly, individuals living with a chronic illness or disability are more likely to search for health information online than those who are healthy (85% vs 61%). The majority of health information seekers search for information every few months or less, primarily around a specific health concern. For those who do not have access to a health care provider, information and treatment resources on the Internet may represent their only contact with the health care system. These data paint a clear and promising picture of a strong market demand for accessible health information.

### Individual Level Behavior Change

Tailored print materials [15-18] and interactive behavior change programs [19] have been shown to have modest efficacy compared to more intensive, clinical programs. However, given the increasing penetration of the Internet in the United States (68.3% as of December 2004 [20]), delivery of such interventions via the Internet (mass customization) can reach much larger numbers of individuals than clinical trials, ultimately affecting population impact (impact = reach × efficacy [21]). Much work has been done to translate proven clinic-based interventions into more broadly available programs. However, the knowledge base in tailored and interactive behavior change programs has not been rigorously tested within the unique context of the Internet. It is critically important that interventions are evaluated within the dissemination context within which they will be used because interventions evaluated in clinical settings or in other modalities (eg, print) may not generalize to the Internet. 

### Systems Level Behavior Change

The Internet and related data management systems can also be used at higher organizational levels to impact the broader socioenvironmental context within which individual cancer prevention behaviors occur. For example, Internet-based systems can be used to conduct assessment and surveillance within communities, worksites, or schools; to evaluate baseline provision of best practice services; and to track critical targets to achieve cancer prevention and treatment goals in real time. Specific to behavioral risk factors, the Internet can be used to remind primary care physicians to counsel their high-risk patients to change risky behaviors and to get age-appropriate screens done for uterine, breast, prostate, and colorectal cancer. It can produce “report cards” regarding the percentage of hospitals, worksites, schools, and communities that meet minimal standards (eg, HEDIS, JCAHO) for providing behavioral change interventions. The Internet can also be used as a tool to test market new interventions, to conduct qualitative research (eg, focus groups, targeted social marketing research), and to gather program and process utilization data in preliminary research studies (eg, [22]).

### Integrating Internet Approaches Into the Health Care System

Internet technology alone will not, in and of itself, be sufficient to reduce the cancer burden at the population level. Rather, the Internet should be conceptualized as a tool embedded within the *context* of the health care and the public health delivery systems and the direct-to-consumer marketing movement. The “Push-Pull” model for translating evidence-based health and behavior research into practice put forth by Orleans et al [23,24] provides a useful framework for thinking about the role of the Internet in cancer prevention and control. The model proposes three activities that are crucial to the dissemination of evidence-based care: (1) “push” of science by proving or improving an intervention for wide population use; (2) “pull” for science by boosting market demand for proven interventions; and (3) building the capacity of relevant systems to deliver or implement them. For those who are actively seeking information via the Internet, there is clearly market demand, or “pull,” for tailored, evidence-based interventions that educate and empower consumers. However, for the vast majority of those at high risk (especially those in low socioeconomic and underserved groups) who are not actively seeking information online, the Internet needs to be conceptualized as simply another channel to “push” evidence-based interventions. Internet-based approaches to cancer prevention and control need to be thoughtfully integrated with efforts from third party payers, for-profit ventures, employers, clinicians, and health care and public health practitioners. Although the Internet has great potential to significantly improve public health and reduce the burden of cancer, there are significant challenges that must be overcome before this potential is realized.

## Challenges of the Internet for Cancer Prevention and Control

### Challenge #1: Quality

Despite the clear role that the Internet now plays in the health care system, there are no data on the impact that the thousands of health-related Web sites have had on public health [25]. Few randomized controlled trials of Internet interventions to modify cancer risk factors have been conducted [26-28]. Taking smoking cessation interventions as an example, the field is very much still in its infancy. Several pilot and uncontrolled studies have been conducted [22,29-32].

In addition, the quality of information on the Internet is a concern. The negative impact of online health information that is inaccurate or misleading, difficult to locate, or difficult to understand may be stronger than the positive impact of high-quality, accurate, evidence-based information. Misinformation on the Internet may have serious and wide-ranging negative consequences, including delays in seeking treatment, violations of privacy and confidentiality, and loss of trust in the health care provider [33]. Limited time with a health care provider may be used inefficiently or unproductively discussing misinformation, ineffective therapies may be chosen over evidence-based treatments, and money may be wasted on sham products and services.

Indeed, numerous studies have documented inadequate coverage of key content areas across a variety of health websites [34-44]. In a recent review of tobacco cessation websites, Bock et al [45] found that of 246 cessation-related websites, only 46 provided actual cessation treatment, and only 5 of those received high ratings for content and usability based on evidence guidelines. The authors concluded that smokers who search the Internet for cessation assistance are unlikely to find high-quality, evidence-based treatment resources. Given that more than 5.5 million smokers search the Internet for information to quit smoking each year [14], and that many arrive at cessation sites when they are most ready to make a quit attempt [22], this is truly a missed opportunity for tobacco control.

#### Methods for Determining Quality

Unfortunately, consumers have few tools at their disposal to determine the quality of information that they find online. According to the Pew Internet & American Life Foundation [46], consumers judge the quality of a website based on whether information is consistent with prior health beliefs, whether information is repeated on multiple sites, whether a site appears commercially driven, and whether the source of the content is available. Numerous measures and tools to evaluate the quality of health information have been developed or proposed (see [33]), including accreditation by an independent entity, rating systems, the use of various seals or logos (eg, HON Code seal), and disclosure of key information about a site. However, none of these methodologies have been applied in any systematic fashion to health behavior change websites. Until there is consensus regarding an appropriate methodology to monitor online content, consumers of online health information are forced to rely on available information to determine quality and trustworthiness of what they read. Thus, disclosure of key information by health websites is vital to empowering consumers to make accurate judgments about quality. Six criteria for rating quality have been proposed by the Commission of the European Communities [47]:

1. **Transparency and honesty:** The provider, purpose, target audience, and funding of the site should be easily identifiable.
2. **Authority:** The source of information should be clear, including credentials of all authors.
3. **Privacy and data protection:** The privacy and data protection policy should be clearly defined.
4. **Updating of information:** Information should be regularly updated to ensure relevance.
5. **Accountability:** Oversight of the website, relationships with partner sites, and selection of content should be held to the highest standards.
6. **Accessibility:** Guidelines on physical accessibility and usability should be followed.

### Challenge #2: Evaluation Models and Methods

In addition to these six criteria for rating quality, we believe a seventh dimension should be added—effectiveness. *Effectiveness* is the effect of information and treatment resources on desired behavioral and/or health outcomes. To date, scientific evaluations of behavior change programs on the Internet reveal no uniform reporting standards regarding effectiveness. While standards exist for evaluating behavioral and pharmacological clinical trials (eg, CONSORT [48], QUORUM [49]), such guidelines have yet to be developed for the specific outcomes evaluation requirements needed for Internet programs [50,51]. One challenge in developing such guidelines is that Internet programs are inherently at the interface between clinical trials research and larger scale dissemination and community demonstration projects, each of which has its own set of guidelines for conducting program, process, and outcomes evaluations. Flay [52] defines an efficacy trial as a well-controlled test of an effect under ideal conditions, which is compared with an effectiveness trial that studies the strength of an intervention effect under real-world conditions. The vast majority of outcomes research to date has been limited to research models based on drug development, such as testing pharmacological and behavior change interventions in small-scale randomized clinical trials under ideal conditions with highly motivated, educated, and self-selected volunteers (clinical efficacy trials). Clinical trials typically focus on initial efficacy in a randomized controlled study conducted with a relatively small sample of a larger target audience. The emphasis is largely on internal validity. Even if multiple clinical trials are conducted, it is still difficult if not impossible to estimate the potential impact of the intervention when adapted and delivered to the whole target population. In contrast, dissemination and community demonstration projects focus on effectiveness of programs when implemented in real-world settings with large target populations. The emphasis is on external validity and the degree to which programs can reach an intended audience. Given the unique ability of Internet programs to bridge basic, clinical, and dissemination research, evaluation standards that are specifically designed for Internet behavior change programs and that balance tensions between internal and external validity need to be developed.

#### The RE-AIM Model

The RE-AIM model of Glasgow et al [53,54] provides a useful model for moving from translational to dissemination research and implementation. Briefly, the RE-AIM framework focuses on five dimensions for evaluating public health interventions: reach, efficacy/effectiveness, adoption, implementation, and maintenance. The RE-AIM framework was designed to address aspects of both internal and external validity that are important in the translation of research to practice [21,55,56]. Reach is defined as the percent of potentially eligible individuals who participate in the intervention study, and how representative they are of the target population from which they are drawn. Efficacy/effectiveness is the intended positive impact of the intervention and its possible unintended consequences on quality of life and related factors. Reach and efficacy/effectiveness operate at the individual level. Adoption is the percent of potential settings and intervention agents that participate in a study and how representative they are of targeted settings/agents. Implementation refers to the quantity and quality of delivery of the intervention's various components. Adoption and implementation are setting-level dimensions. Finally, the maintenance dimension includes individual- and setting-level indices. At the individual level, maintenance is defined as the longer term efficacy/effectiveness of an intervention. Outcomes at 6 months post-intervention contact reflect longer term individual maintenance. The setting-level definition of maintenance refers to the institutionalization of a program and is assessed according to the percent of settings that continue the intervention program, in part or in whole, beyond the study duration [53,54]. The RE-AIM framework forms a useful heuristic to guide the field by the general principles needed to achieve a successful dissemination research knowledge base for a mode of intervention delivery such as the Internet.

More rigorous dissemination research is essential if the full potential of Internet lifestyle change programs is to be realized. At the present time there are few studies of dissemination that address the criteria specified in the RE-AIM model. Dzewaltowski et al [57] reviewed 27 community-based dissemination intervention studies that “promoted good nutrition, physical activity or smoking cessation/prevention” and evaluated the extent to which each study reported on elements of the RE-AIM model. Although most studies (88%) reported participation rates among eligible members of the target audience (“reach”), only 11% of studies reported the participation rate (“adoption”) among eligible organizations or settings. Even fewer studies reported if participation was representative of those found in the broader population. Although 59% of studies reported whether the intervention was delivered (“implementation”), few reported whether individuals maintained the behavior change (30%) or whether organizations institutionalized interventions (0%). The authors concluded that “…to increase the potential to translate community research findings to practice, studies should place a greater emphasis on obtaining and reporting external validity information, such as representativeness” [57].

#### Application of RE-AIM to Internet Research

Many of the emergent challenges to conducting community-based dissemination research in general also apply to Internet-based research specifically. Dzewaltowski et al [57] recommend that dissemination studies include “a comparison of the study sample with either the broader target population or with those that decline, with respect to basic demographic data (Reach). This comparison can often be made using available datasets (eg, census data). Where such data is unavailable, researchers should attempt to gather basic demographic data on all participants contacted for recruitment and subsequently compare those that agreed to participate with those that declined” [57] (p. 242). They also recommend “that researchers record the level of fidelity with which the intervention is delivered (Implementation). This evaluation should include how much of the intervention protocol was followed as intended, the timeliness of protocol implementation, and any adaptations of the intervention protocol (ie, any deviations from a treatment protocol developed in an ideal clinical trial context)” [57] (p. 243).

#### The Need for Standards

The need for a new and broader set of standards for dissemination research trials in general and for Internet programs in particular presents a formidable challenge to the field. In considering the criteria for these standards, a balance needs to be found between preserving internal validity and maximizing external validity. On the one hand, the best research designs and methods derived from clinical trials research guidelines (eg, CONSORT) need to be retained where feasible. On the other hand, evaluating interventions as they are being used in the real world may require methods other than randomized controlled trials (eg, [58]). Despite the daunting challenges, we recommend that specific criteria be developed for reporting results of program, process, and outcomes evaluation of Internet programs. These criteria should specify the minimal acceptable standards of evidence for success, building on guidelines like the CONSORT criteria and others that have advanced the evidence base by improving the rigor of clinical trials. Standard methods of reporting the population parameters of dissemination research are needed to create a level playing field in order to make meaningful comparisons between intervention studies. For example, to define the “Reach” of an intervention, all study “denominators” should be documented, starting with the entire defined population from which the participants were drawn.

## A Transdisciplinary Science Approach

There are numerous conceptual and practical issues and various perspectives at different levels of analysis that must be integrated to address the many challenges outlined thus far in this paper. To speed the development and evaluation of evidence-based Internet interventions, we recommend that a team approach to research be adopted to (a) encourage a coordinated and more rapid shift from basic to clinical trials to dissemination research; (b) capitalize on the Internet's real time tracking capability to enhance basic research (treatment components, mediators, and moderators) and to link program and process evaluation to outcomes; (c) involve practitioners, policy makers, other stakeholders, and business leaders in the research process (eg, incorporating a business model within a rigorous research framework); and (d) involve consumers so that the end users of programs are included from the very outset of the development and evaluation in order to ensure credibility, marketability, and utility. Another key to successful dissemination research is the team approach used in models such as “practice based networks” [59] and action research strategies [60]. The movement towards transdisciplinary science in fields that cut across traditional boundaries appears to be particularly applicable to address the challenges in development and evaluation of Internet behavior change interventions.

We believe that transdisciplinary science is a way to address the challenges in harnessing the potential of the Internet for cancer prevention and control. In defining transdisciplinary science, Rosenfield [61] made the following distinctions:

- **Multidisciplinarity** refers to a process in which researchers in different disciplines work relatively independently, each from his or her own disciplinary perspective with limited direct interaction and little cross-fertilization among disciplines.
- **Transdisciplinarity** is a process by which collaborators work jointly on a common problem from the very outset, using a shared conceptual framework that draws together discipline-specific theories, methods, and measures into a new synthesis.

Transdisciplinary research involves joint, coordinated, and continuously integrated research done by experts with different disciplinary backgrounds, working together and producing joint reports, papers, recommendations, and plans. Ideas from each participant are so thoroughly interwoven that the specific contributions of each participant tend to be obscured by the joint product. Early hallmarks of transdisciplinary science are the development of new approaches to theory, design, methods, measurement, and data analysis.

A transdisciplinary framework is needed because the challenges facing Internet research cannot be readily resolved by any one scientific discipline, group of stakeholders, or methodological approach to evaluation. A transdisciplinary approach recognizes that increasingly complex problems such as evaluating Internet behavioral interventions require a “team science” solution. The potential success of Internet behavior change programs is compromised at this time because the various groups involved in the design and delivery of such programs (eg, basic and applied scientists, health care providers, insurers, entrepreneurs, consumers, other stakeholders) have generally not collaborated in all phases of program development. As a result, there is great variability in the quality of existing Internet programs in terms of content, usability, and outcomes evaluation data.

The Internet offers unique opportunities to the transdisciplinary team to advance theory and to understand the basic mechanisms that lead to successful behavior change. The technological capabilities of the Internet permit a fine-grained collection of a wide variety of information and measures over time. In typical clinical trials or dissemination research, such detailed levels of tracking in real time are virtually impossible. In contrast, it is possible to track which specific components of an Internet intervention are used by each individual as well as the intensity of use. In addition, tracking of utilization data can be done across thousands of users, and the data can be automatically stored and “mined” at little or no additional cost. Metrics such as number of log-ins, total time spent online, average time per session, and number of page views are some of the more basic methods of establishing whether an intervention was delivered and received as intended (ie, internal validity). The mediators and moderators of successful or poor outcomes can also be analyzed. For example, researchers can determine what proportion of participants used a specific feature of an Internet program, what cognitive or behavioral factors changed as a result of program use, and which particular participants benefited most. Perhaps of greatest interest is the opportunity to link treatment utilization data with behavioral outcomes. An example of this type of analysis would be examining to what extent the intensity of online social support (eg, total time spent in a chat room, number of bulletin board posts in a one-month period) is related to a desired outcome (eg, changes in perceived quality of life, increases in physical activity).

## Case Example: Online Social Support for Smoking Cessation

To illustrate the capability of the Internet to advance theory, we present a brief example from our research on the role of Internet-based social support in smoking cessation [22]. It is well established that the social environment plays an important role in smoking cessation. High levels of social support have been related to better cessation outcomes in clinical trials [7]. However, attempts to enhance the effectiveness of smoking cessation interventions by manipulating social support have achieved only modest success in most smoking cessation clinical trials [62]. Experimental manipulations of social support have included interventions designed to create new social networks, to train smokers to influence their own networks, or to train network members to be more supportive of the smoker. One reason for this modest success is that a “critical mass” of diverse, accessible, and anonymous sources of social support is simply not available in clinical settings where treatment is delivered to individuals or small groups on a weekly basis. We were interested in determining if perceptions of support and the use of online “support services” (eg, email, chat rooms, ask an expert) were associated with improved cessation outcomes among users of a broadly disseminated, evidence-based [45] smoking cessation website (QuitNet). Our interest in the construct of social support on the Internet derives from the thriving and naturalistic occurrence of Internet-based social support on this website.

### Evaluation of QuitNet

We conducted a large-scale, preliminary evaluation of QuitNet (see [22] for details). Consecutive registrants to the QuitNet site (N = 1501) were surveyed 3 months after they registered in order to assess 7-day point prevalence abstinence. Process-to-outcome analyses indicated that the use of social support was associated with more than three times greater point prevalence abstinence, and more than four times greater continuous abstinence [22]. Not surprisingly, those who were quit at follow-up participated more extensively in the various opportunities for social support than those who were still smoking. It is noteworthy that baseline motivation was not significantly correlated with website use (intensity, use of social support) or with smoking outcomes.

We also examined whether greater duration and frequency of treatment (ie, intensity) was associated with better cessation outcomes as reported in the US Public Health Service guideline [7]. Using logistic regression with a post hoc median split of “high” vs “low” intensity website use as the predictor, analyses indicated that high website users were more than twice as likely to be continuously abstinent for 2 months compared to low website users. A composite measure of website utilization intensity (number of log-ins × duration in minutes per log-in) was very highly correlated with use of support resources (number of emails sent, number of emails received, number of email senders, number of email recipients). Since intensity and social support predicted cessation outcomes, and since social support increases with intensity, we then examined whether the degree of social support mediated the effect of intensity on cessation [63]. Confirming the mediation hypothesis requires that the effect of intensity be attenuated after adjusting for the effect of social support in a regression analysis. Indeed, mediation was found with smoking cessation as the outcome: the odds ratio for the effect of intensity declined from 2.34 to 1.52 after adding social support to the model [22]. On the other hand, high social support continued to almost triple the odds of quitting relative to low social support even after adjusting for intensity of website use (see [22] for more details).

### Future Directions for Internet Behavior Change Research

The brief case example illustrates how the Internet can provide a platform to test theories of how social support may be used to enhance behavior change and maintenance of behavior change. Indeed, the Internet provides the tools for fine-grained data collection from large numbers of participants as they interact over time. Simply studying the natural emergence and evolution of Internet-based support groups may even provide opportunities to develop new theories and measures (see below) of how different kinds of social support systems motivate and mediate behavior change for different types of users at different times during the change process.

Another advantage of the Internet for advancing basic science is that new methods can be applied to the massive amounts of available data. For instance, patterns and content of online interactions between and among individuals participating in smoking cessation chat rooms can be analyzed using qualitative and quantitative analytic methods. Pennebaker et al have used innovative techniques derived from psycholinguistics and other disciplines to conduct studies of Internet and real-world support groups for 20 different diseases [64]. They also have described an analysis of over 1000 people who wrote online journals in the weeks before and after the terrorist attacks on September 11th [65]. Perhaps these techniques can be applied to understand how Internet social support helps smokers to quit, to maintain abstinence, and to prevent relapse.

The transdisciplinary science team must not only build on what is already known but must also develop new conceptual models that enhance the goals of maximizing the specific mode of Internet-delivered programs. Specific challenges include understanding how to best (a) reach, attract, and retain consumers in using appropriate (ie, evidence based) websites; (b) facilitate initial behavior change and provide knowledge and skills for successful change; (c) enhance consumer motivation to engage in behavior change activities over time; and (d) ensure that initial change is maintained. The Internet may not only be a tool for large-scale dissemination research, but it may also be useful for advancing theories of behavior change and developing new theories and ways to improve future Internet interventions.

## Conclusions

There is great potential for the Internet to impact cancer prevention and control. The Internet can be used to promote lifestyle change across the cancer continuum, from primary prevention to treatment to survivorship. In addition, high-quality, evidence-based information and treatment resources can empower individuals, families, and communities to become educated consumers, to become active in their own preventive health care, and to demand more of the health care system with regard to health promotion and disease prevention. For this to happen, Internet interventions are needed that are known to be efficacious, low cost, accessible, sustainable, and that can reach large target populations. Policy makers, practitioners, and the general public cannot wait until definitive evidence regarding behavior change programs for delivery via the Internet is available. A consumer-driven thirst for health information is currently being met by the Internet with its myriad websites, many of poor quality. Scientific experts and public health practitioners must provide consumers with tools to find and use high-quality information and evidence-based treatment programs on the Internet. New transdisciplinary research domains are needed that bridge the basic, clinical, public health, and policy arenas, placing special emphasis on dissemination research. The science to practice gap must be closed to integrate basic mechanism research with translational and dissemination outcomes research for delivery of health information via the Internet. Critical to the successful emergence of better practices is the need to communicate to consumers the latest information about the quality, credibility, usability, and content of programs available on the Internet. Together, new technology in informatics and a transdisciplinary approach to product development and evaluation can improve the quality and cost-effectiveness of behavior change programs in order to reduce the burden of cancer.

## Abbreviations

CONSORT: Consolidated Standards of Reporting Trials
HEDIS: Health Plan Employer Data and Information Set
JCAHO: Joint Commission on Accreditation of Healthcare Organizations
QUORUM: Quality of Reporting of Meta-Analyses

## References

[1] Miniño Arialdi M, Arias Elizabeth, Kochanek Kenneth D, Murphy Sherry L, Smith Betty L (2002). Deaths: final data for 2000. Natl Vital Stat Rep.

[2] American Cancer Society (2004). Cancer facts & figures 2004.

[3] Miller Suzanne M, Bowen Deborah J, Campbell Marci K, Diefenbach Michael A, Gritz Ellen R, Jacobsen Paul B, Stefanek Michael, Fang Carolyn Y, Lazovich DeAnn, Sherman Kerry A, Wang Catharine (2004). Current research promises and challenges in behavioral oncology: report from the American Society of Preventive Oncology annual meeting, 2002. Cancer Epidemiol Biomarkers Prev.

[4] Hiatt R A, Rimer B K (1999). A new strategy for cancer control research. Cancer Epidemiol Biomarkers Prev.

[5] US Dept of Health and Human Services (2003). Behavioral Risk Factor Surveillance System (BRFSS) - Public use data tape 2002.

[6] Curry Susan J, Byers Tim, Hewitt Maria Elizabeth (2003). Fulfilling the Potential of Cancer Prevention and Early Detection.

[7] The Tobacco Use and Dependence Clinical Practice Guideline Panel, Staff, and Consortium Representatives (2000). A clinical practice guideline for treating tobacco use and dependence: A US Public Health Service report. JAMA.

[8] Task Force on Community Preventive Services (2002). Recommendations to increase physical activity in communities. Am J Prev Med.

[9] Saraiya Mona, Glanz Karen, Briss Peter, Nichols Phyllis, White Cornelia, Das Debjani, Task Force on Community Preventive Services On reducing Exposure to Ultraviolet Light (2003). Preventing skin cancer: findings of the Task Force on Community Preventive Services On reducing Exposure to Ultraviolet Light. MMWR Recomm Rep.

[10] U.S. Preventive Services Task Force (2004). Screening and behavioral counseling interventions in primary care to reduce alcohol misuse: recommendation statement. Ann Intern Med.

[11] Mcglynn Elizabeth A, Asch Steven M, Adams John, Keesey Joan, Hicks Jennifer, Decristofaro Alison, Kerr Eve A (2003). The quality of health care delivered to adults in the United States. N Engl J Med.

[12] Committee on Quality Health Care in America (2003). Crossing the Quality Chasm: A New Health System for the 21st Century.

[13] Lenhart A, Horrigan J, Rainie L The ever-shifting Internet population: a new look at Internet access and the digital divide.

[14] Fox S, Fallows D Internet health resources: health searches and email have become more commonplace, but there is room for improvement in searches and overall Internet access.

[15] Rimer B K, Glassman B (1999). Is there a use for tailored print communications in cancer risk communication?. J Natl Cancer Inst Monogr.

[16] Dijkstra A, De Vries H (1999). The development of computer-generated tailored interventions. Patient Educ Couns.

[17] Strecher V J (1999). Computer-tailored smoking cessation materials: a review and discussion. Patient Educ Couns.

[18] Abrams D B, Mills S, Bulger D (1999). Challenges and future directions for tailored communication research. Ann Behav Med.

[19] Glasgow Russell E, Bull Sheana S, Piette John D, Steiner John F (2004). Interactive behavior change technology. A partial solution to the competing demands of primary care. Am J Prev Med.

[20] Internet World Stats Usage and Population Statistics.

[21] Abrams D, Orleans C, Niaura R (1996). Integrating individual and public health perspectives for treatment of tobacco dependence under managed health care: a combined stepped care and matching model. Ann Behav Med.

[22] Cobb N, Graham AL, Bock B, Papandonatos GC, Abrams DB Initial evaluation of a 'real-world' Internet smoking cessation system. Nicotine Tob Res.

[23] Orleans CT, Gruman J, Anderson N Roadmaps for the next frontier: getting evidence-based behavioral medicine into practice.

[24] Gruman Jessie C, U.S. Department of Health and Human Services (1998). Putting evidence into practice: the OBSSR report of the Working Group on the Integration of Effective Behavioral Treatments into Clinical Care.

[25] Coiera E (1998). Information epidemics, economics, and immunity on the internet. We still know so little about the effect of information on public health. BMJ.

[26] Marshall Alison L, Leslie Eva R, Bauman Adrian E, Marcus Bess H, Owen Neville (2003). Print versus website physical activity programs: a randomized trial. Am J Prev Med.

[27] Womble Leslie G, Wadden Thomas A, Mcguckin Brian G, Sargent Stephanie L, Rothman Rebecca A, Krauthamer-ewing E Stephanie (2004). A randomized controlled trial of a commercial internet weight loss program. Obes Res.

[28] Irvine A Blair, Ary Dennis V, Grove Dean A, Gilfillan-morton Lynn (2004). The effectiveness of an interactive multimedia program to influence eating habits. Health Educ Res.

[29] Lenert Leslie, Muñoz Ricardo F, Stoddard Jackie, Delucchi Kevin, Bansod Aditya, Skoczen Steven, Pérez-stable Eliseo J (2003). Design and pilot evaluation of an internet smoking cessation program. J Am Med Inform Assoc.

[30] Lenert Leslie, Muñoz Ricardo F, Perez John E, Bansod Aditya (2004). Automated e-mail messaging as a tool for improving quit rates in an internet smoking cessation intervention. J Am Med Inform Assoc.

[31] Stoddard JL, Munoz RF, Delucchi KL A smoking cessation feasibility study conducted over the World Wide Web. J Health Commun.

[32] Feil Edward G, Noell John, Lichtenstein Ed, Boles Shawn M, Mckay H Garth (2003). Evaluation of an Internet-based smoking cessation program: lessons learned from a pilot study. Nicotine Tob Res.

[33] Eng TR (2001). The eHealth Landscape: A Terrain Map of Emerging Information and Communication Technologies in Health and Health Care.

[34] Maloney S, Ilic D, Green S (2005). Accessibility, nature and quality of health information on the Internet: a survey on osteoarthritis. Rheumatology (Oxford).

[35] England C Y, Nicholls A M (2004). Advice available on the Internet for people with coeliac disease: an evaluation of the quality of websites. J Hum Nutr Diet.

[36] Ilic Dragan, Risbridger Gail, Green Sally (2004). Searching the Internet for information on prostate cancer screening: an assessment of quality. Urology.

[37] Kunst Heinke, Khan Khalid S (2002). Quality of web-based medical information on stable COPD: comparison of non-commercial and commercial websites. Health Info Libr J.

[38] Cheh Julie A, Ribisl Kurt M, Wildemuth Barbara M (2003). An assessment of the quality and usability of smoking cessation information on the Internet. Health Promot Pract.

[39] Berland G K, Elliott M N, Morales L S, Algazy J I, Kravitz R L, Broder M S, Kanouse D E, Muñoz J A, Puyol J A, Lara M, Watkins K E, Yang H, Mcglynn E A (2001). Health information on the Internet: accessibility, quality, and readability in English and Spanish. JAMA.

[40] Anselmo Mark A, Lash Katherine M, Stieb Elisabeth S, Haver Kenan E (2004). Cystic fibrosis on the Internet: a survey of site adherence to AMA guidelines. Pediatrics.

[41] Hoffman-Goetz L, Clarke J N (2000). Quality of breast cancer sites on the World Wide Web. Can J Public Health.

[42] Butler Laura, Foster Nadine E (2003). Back pain online: a cross-sectional survey of the quality of web-based information on low back pain. Spine.

[43] Bichakjian Christopher K, Schwartz Jennifer L, Wang Timothy S, Hall Janette M, Johnson Timothy M, Biermann J Sybil (2002). Melanoma information on the Internet: often incomplete-a public health opportunity?. J Clin Oncol.

[44] Eysenbach Gunther, Powell John, Kuss Oliver, Sa Eun-Ryoung (2002). Empirical studies assessing the quality of health information for consumers on the world wide web: a systematic review. JAMA.

[45] Bock Beth, Graham Amanda, Sciamanna Christopher, Krishnamoorthy Jenelle, Whiteley Jessica, Carmona-barros Rosa, Niaura Raymond, Abrams David (2004). Smoking cessation treatment on the Internet: content, quality, and usability. Nicotine Tob Res.

[46] Fox S, Rainie L (2002). Vital Decisions: How Internet users decide what information to trust when they or their loved ones are sick.

[47] Commission of the European Communities, Brussels (2002). eEurope 2002: Quality Criteria for Health Related Websites. J Med Internet Res.

[48] Moher D (1998). CONSORT: an evolving tool to help improve the quality of reports of randomized controlled trials. Consolidated Standards of Reporting Trials. JAMA.

[49] Moher D, Cook D J, Eastwood S, Olkin I, Rennie D, Stroup D F (1999). Improving the quality of reports of meta-analyses of randomised controlled trials: the QUOROM statement. Quality of Reporting of Meta-analyses. Lancet.

[50] Eysenbach Gunther (2002). Issues in evaluating health websites in an Internet-based randomized controlled trial. J Med Internet Res.

[51] Gosling Samuel D, Vazire Simine, Srivastava Sanjay, John Oliver P (2004). Should we trust web-based studies? A comparative analysis of six preconceptions about internet questionnaires. Am Psychol.

[52] Flay B R (1986). Efficacy and effectiveness trials (and other phases of research) in the development of health promotion programs. Prev Med.

[53] Glasgow R E, Vogt T M, Boles S M (1999). Evaluating the public health impact of health promotion interventions: the RE-AIM framework. Am J Public Health.

[54] Dzewaltowski David A, Glasgow Russell E, Klesges Lisa M, Estabrooks Paul A, Brock Elizabeth (2004). RE-AIM: evidence-based standards and a Web resource to improve translation of research into practice. Ann Behav Med.

[55] Rogers Everett M (1995). Diffusion of Innovations.

[56] Oldenburg B F, Sallis J F, Ffrench M L, Owen N (1999). Health promotion research and the diffusion and institutionalization of interventions. Health Educ Res.

[57] Dzewaltowski David A, Estabrooks Paul A, Klesges Lisa M, Bull Sheana, Glasgow Russell E (2004). Behavior change intervention research in community settings: how generalizable are the results?. Health Promot Int.

[58] Tunis Sean R, Stryer Daniel B, Clancy Carolyn M (2003). Practical clinical trials: increasing the value of clinical research for decision making in clinical and health policy. JAMA.

[59] Glasgow Russell E, Goldstein Michael G, Ockene Judith K, Pronk Nicolaas P (2004). Translating what we have learned into practice. Principles and hypotheses for interventions addressing multiple behaviors in primary care. Am J Prev Med.

[60] Abrams DB, Emmons KM, Linnan LA, Glanz Karen, Rimer Barbara K, Lewis Frances Marcus (1997). Health behavior and health education: The past, present, and future. Health Behavior and Health Education: Theory, Research, and Practice.

[61] Rosenfield P L (1992). The potential of transdisciplinary research for sustaining and extending linkages between the health and social sciences. Soc Sci Med.

[62] Lichtenstein E, Glasgow RE, Abrams DB (1986). Social support in smoking cessation: in search of effective interventions. Behavior Therapy.

[63] Baron R M, Kenny D A (1986). The moderator-mediator variable distinction in social psychological research: conceptual, strategic, and statistical considerations. J Pers Soc Psychol.

[64] Davison K P, Pennebaker J W, Dickerson S S (2000). Who talks? The social psychology of illness support groups. Am Psychol.

[65] Cohn Michael A, Mehl Matthias R, Pennebaker James W (2004). Linguistic markers of psychological change surrounding September 11, 2001. Psychol Sci.

